# Longitudinal association between N-terminal B-type natriuretic peptide, anxiety and social support in patients with HFpEF: results from the multicentre randomized controlled Aldo-DHF trial

**DOI:** 10.1186/s12872-023-03136-8

**Published:** 2023-04-05

**Authors:** Marieke R. Wilke, Daniel Broschmann, Anja Sandek, Rolf Wachter, Frank Edelmann, Christoph Herrmann-Lingen

**Affiliations:** 1 Department of Psychosomatic Medicine and Psychotherapy, University of Göttingen Medical Center, Von-Siebold-Str. 5, 37075 Göttingen, Germany; 2 Department of Cardiology, University Hospital Leipzig, Liebigstr. 20, Haus 4, 04103 Leipzig, Germany; 3 Clinic for Cardiology and Pneumology, University of Göttingen Medical Center, Robert-Koch-Straße 40, 37075 Göttingen, Germany; 4 German Center for Cardiovascular Research (DZHK), Partner Site Göttingen, Robert-Koch-Straße 40, 37075 Göttingen, Germany; 5 Department of Internal Medicine and Cardiology, Campus Virchow Klinikum, Charité Universitätsmedizin Berlin, Mittelallee 11, 13353 Berlin, Germany; 6 German Center for Cardiovascular Research, Partner Site Berlin, Robert-Rössle-Str. 10, 13125 Berlin, Germany

**Keywords:** Anxiety, HFpEF, NT-proBNP, Social support

## Abstract

**Background:**

Higher plasma levels of natriuretic peptides (NPs) have been associated with reduced anxiety in experimental research and a number of patient samples. As NP levels are elevated in heart failure patients, we investigate whether this elevation is related to anxiety in patients with heart failure with preserved ejection fraction (HFpEF).

**Methods:**

Post-hoc regression and mediation analyses were conducted, using data of 422 patients with HFpEF from the randomized, placebo-controlled, double-blinded, two-armed, multicentre aldosterone in diastolic heart failure trial, testing associations and their mediators between the N-terminal B-type natriuretic peptide (NT-proBNP) and anxiety at baseline and over 12-month follow-up. Anxiety was measured by the Hospital Anxiety and Depression Scale (HADS), social support by the ENRICHD Social Support Inventory and physical functioning by the Short Form 36 Health Survey.

**Results:**

The mean age of the study population was 66.8 ± 7.6 years, 47.6% were male and 86.0% had NYHA class II. NT-proBNP showed a weak negative correlation with HADS anxiety scores at baseline (r = − 0.087; p = 0.092), which was significant (r = − 0.165; p = 0.028) in men but not in women. NT-proBNP also tended to predict lower anxiety at 12-months in men. On the other hand, higher anxiety at baseline was associated with lower NT-proBNP scores 12 months later (r = − 0.116; p = 0.026). All associations lost significance in multivariate regression for age, perceived social support (ESSI), physical function (SF-36) and study arm. Mediation analyses revealed that social support acts as a full mediator for the link between NT-proBNP levels and anxiety.

**Conclusion:**

The mechanisms linking NT-proBNP to anxiety may be more complex than originally assumed. While effects of NT-proBNP on anxiety may be mediated by perceived social support, there may be an additional negative effect of anxiety on NT-proBNP. Future research should consider this possible bi-directionality of the association and assess the potential influence of gender, social support, oxytocin and vagal tone on the interaction of anxiety and natriuretic peptide levels.

*Trial Registration* http://www.controlled-trials.com (ISRCTN94726526) on 07/11/2006.

Eudra-CT-number: 2006–002,605-31.

## Background

Heart failure is a leading cause of morbidity and mortality and its increasing prevalence places an enormous burden on the health care system and society [1, 2]. Epidemiologic studies indicate that up to half of patients with heart failure have a left ventricular ejection fraction (LVEF) of at least 50%, which is referred to as heart failure with preserved left ventricular ejection fraction (HFpEF). However, there are considerably fewer data on its etiology and therapy compared to heart failure with reduced LVEF (HFrEF). Furthermore, a growing body of research points to a mutual deterioration of mental health problems and cardiovascular diseases [3–5]. Patients with heart failure show an increased prevalence of reported anxiety. In this population the data vary between 6 and 72% with an average prevalence of 32%. However, prevalence partly depends on the respective measurement instrument and definition of elevated anxiety [6]. Consideration of comorbid anxiety disorders may be of prognostic value as they may increase the risk of developing or aggravating heart failure and are linked to adverse medical and functional outcomes [7, 8].

Natriuretic peptides secreted in response to increased cardiac stress are widely used indicators of cardiac disease severity. Consequently, one might expect higher levels of these peptides to be associated with an increase in psychological distress, including more symptoms of anxiety or depression. Interestingly though, a growing body of research indicates that higher plasma levels of these peptides may be associated with better mental health [9].

### Natriuretic peptides and mental health

Through their effect on renal, cardiovascular, neuronal and endocrine functions, natriuretic peptides such as atrial natriuretic peptide (ANP) and B-type natriuretic peptide (BNP) play a crucial role in the regulation of blood pressure and fluid balance [10]. During BNP secretion, the precursor peptide ProBNP is equimolarly cleaved into BNP and the biologically inactive NT-proBNP (amino-terminal-cleavage-fragment of BNP). Since the latter is eliminated exclusively renally and has a higher concentration and longer half-life in plasma/serum than BNP, it has established itself as the preferred measurement value compared to BNP.

Interestingly, they seem to be associated with anxiety as well. For example, Herrmann-Lingen et al. [11] found a negative correlation between pro-ANP levels and self-assessed anxiety in a mixed sample of patients with cardiovascular risk factors or diagnosed heart failure. In the patients from the DIAST-CHF study with risk factors for HFpEF, higher plasma levels of mid-regional proANP were significantly associated with reduced anxiety and for NT-proBNP a trend towards reduced anxiety was observed [12].

### Effects on the HPA axis

One explanatory approach for this possible anxiolytic effect deals with the inhibiting effect on the stress-sensitive hypothalamic–pituitary–adrenal axis (HPA axis) and sympathetic tone, which became apparent in various behavioural studies in rats and in clinical studies with patients suffering from panic disorder [13–17]. In line with these results, an anti-ANP serum administered to rats prior to a stress response significantly increased the stress-induced secretion of Adreno-corticotropic hormone (ACTH) and Corticosterone compared to rats with physiological ANP function [18]. Conversely, in humans, administration of ANP prior to pharmacological induction of a panic attack using cholecystokinin tetrapeptide reduced the occurrence of panic attacks [15].

Although ANP and BNP show many similarities in structure and function, there are considerably fewer study results on psychological effects of BNP, compared to ANP. In patients with coronary heart disease and mild to moderate depressive symptoms Fangauf et al. [19] found a significant negative correlation between NT-proBNP levels and the subjective perception of anxiety, depression, fatigue, physical pain and (poor) mental health, which persisted after multivariate adjustment. Furthermore, in a follow-up analysis, they observed that higher baseline levels of NT-proBNP were associated with persistently lower anxiety scores over 24 months [20].

Based on the literature suggesting a negative correlation between natriuretic peptides and anxiety, our primary hypothesis was that higher NT-proBNP plasma levels are correlated with reduced anxiety in HFpEF patients. Although research to date supports an anxiolytic effect of natriuretic peptides, increased anxiety may, conversely, have a suppressive effect on the peptides’ release or production. As most of the previous findings on this relationship come from exclusively cross-sectional studies, longitudinal data from this study may shed new light on a possible bidirectional association between anxiety and NT-proBNP levels, providing the basis for a better understanding of the underlying mechanisms.

## Methods

### Study design

The Aldosterone Receptor Blockade in Diastolic Heart Failure (Aldo-DHF) trial was a multicenter, prospective, randomized, double-blind, placebo-controlled, two-armed study in patients diagnosed with chronic heart failure stage II or III according to the New York Heart Association (NYHA) and left ventricular ejection fraction > 50%. Additional inclusion criteria were a minimum age of 50 years and echocardiographic evidence of diastolic dysfunction (≥ Grade I) or atrial fibrillation. Main exclusion criteria were various indicators of poor health. The study design has been published previously [21].

Aldo-DHF was conducted in accordance with Good Clinical Practice guidelines and the Declaration of Helsinki. The ethics committees of all participating centers reviewed and accepted the trial protocol and all patients gave their written informed consent before being included in the trial. The trial randomized 422 patients to either 25 mg daily of the aldosterone receptor blocker spironolactone or placebo. Primary endpoints of the study were changes in diastolic left ventricular function and exercise capacity after 12 months.

#### Clinical assessment

Medical history, clinical examination, electrocardiography, echocardiography, laboratory diagnostics, spiroergometry, six-minute walking test and quality of life assessment were performed at baseline and after 6 and 12 months. Blood was drawn from a cubital vein in resting, non-fasting patients and centrifuged ten minutes in an Eppendorf 5702R centrifuge. The plasma thus obtained was pipetted off and stored at − 80 °C until being analyzed. In the case of blood analyses at external sites, the cold chain was checked during the transport of the blood samples with the help of the company B.R.A.H.M.S. AG Hennigsdorf. Plasma concentrations of NT-proBNP were determined centrally at the University of Göttingen Medical Center using the Elecsys® proBNP test from Roche Diagnostics. The test is a non-competitive electrochemiluminescent immunoassay whose specific polyclonal antibodies bind to certain regions of NT-proBNP.

#### Psychosocial assessment

The patients' psychosocial status was assessed using the validated German versions of standardized widely used self-rating questionnaires. Anxiety was assessed by the anxiety subscale of the Hospital Anxiety and Depression Scale (HADS) in its German version [22]. The anxiety subscale (HADS-A) consists of seven items asking for symptoms of generalized anxiety and panic, the frequency or extent of which are assessed during the previous week using a 4-level Likert scale (0–3 points) [23]. For evaluation purposes, the 7 item values are summed up to give the sum value for HADS-A with a value range from 0 to 21. Higher values indicate more anxiety [24]. As a cut-off value, a sum value of 8 yields an optimal balance of sensitivity and specificity [25, 26].

Perceived social support was assessed by the German version [27] of the ENRICHD Social Support Inventory (ESSI). The ESSI was developed for patients after myocardial infarction and asks about their perceived emotional support using a 5-point Likert scale. For evaluation, the assigned item values are summed up, with higher values indicating more social support in the patient's environment. A scale value of ≤ 18 and a scale value of ≤ 3 for at least 2 items corresponds to low social support [28].

The Short Form 36 Health Questionnaire (SF-36) is a 36-item, generic instrument designed to measure health-related quality of life related to the last four weeks. It contains eight subscales, one of which assesses physical function [29]. The individual items are answered using Likert scales and eventually coded into a numerical value from 0 to 100, whereby a higher numerical value corresponds to a better self-rated state of health. Finally, a mean score is calculated from the item scores for each subscale [30]. The German version was validated by Bullinger et al. [31].

#### Data analysis

The statistical analyses were performed with IBM SPSS Statistics Versions 25–28 and the significance level was set at 5% (2-sided). Missing values were excluded pairwise for correlation analyses and listwise for regression and mediation analyses.

Normality was tested using the Kolmogorov–Smirnov and Shapiro–Wilk test. As the distribution of NT-proBNP values was severely skewed, they were log-transformed for tests that require normality. The results of the power analysis for the Aldo-DHF study were reported in the previously published study design.

Demographic and clinical data are presented as means ± standard deviations, median and interquartile range or frequencies and percentages, as appropriate. We used bivariate correlations according to Pearson to evaluate the association between log-transformed values of the biomarker NT-proBNP and scores of the subscale "anxiety" of the HADS at baseline and at 12 months. To identify potentially confounding variables, we tested perceived social support (ESSI), age, BMI and objective severity markers of the physical disease (LVEF, E/A ratio, 6-min walking distance, peak VO2). Of these, social support significantly correlated with HADS anxiety and NT-proBNP at baseline and at 12 months and was therefore included in the respective regression models. All multivariate regression models were adjusted for age and in the full cohort additionally for sex as a standard. The longitudinal regression models were adjusted for study arm to test for potential impact of treatment with spironolactone. Finally, we adjusted for SF-36 Physical function as a measure of perceived disease severity to disentangle the potential relevance of two opposite processes: On the one hand, the literature suggests that BNP may have an anxiety-relieving effect. On the other hand, (NT-pro)BNP reflects increased cardiac stress, indicating more severe disease, which can in turn be expected to increase anxiety.

Based on recent data from Fangauf et al. [20] suggesting a sex difference in the association of NT-proBNP and anxiety we repeated the analyses separately for men and women.

Mediation analyses for social support were performed using the bias-corrected bootstrap confidence interval test [32]. Unstandardized indirect effects were computed for each of 1000 bootstrapped samples and significance was tested by means of 95% confidence intervals (CI). The total effect is the effect of x on y without the mediator. The direct effect is the effect of x on y with the mediator in the model and the indirect effect is the actual mediation effect. A mediation effect is likely involved if the total and direct effect is not significant and the indirect effect is significant.

## Results

### Descriptive statistics

The ALDO-DHF study enrolled 422 patients with an average age of 66.8 years, a mean E/A ratio of 12.8 and a BMI of 28.9 kg/m^2^. The average systolic blood pressure was 135 mmHg and the maximum oxygen uptake (peak VO2) was 16.4 ml/kg/min. Patients had a median NT-proBNP value of 158.4 pg/ml and an average HADS anxiety score of 5.3. The majority of patients was classified as NYHA II (86.2%) and diagnosed with hypertension (91.7%). HADS anxiety, social support and median NT-proBNP plasma levels did not significantly differ between female and male patients. Women showed a significantly higher E/A ratio, a lower systolic blood pressure and better physical functioning on the SF-36. Men were more likely to have CAD or diabetes mellitus (Table 1).

**Table 1:** Baseline characteristics of the patient population with differentiation according to sex

Variable		Whole sample	Sex difference	
			Male N = 201 (47.6%)	
			Female N = 221 (52.4%)	
Age [Y]	M (SD)	66.8 (7.6)	Male	66.5 (7.7)
			Female	67.1 (7.5)
NYHA class II	N (%)	363 (86.2)	Male	185 (51) **
			Female	178 (49)
NYHA class III	N (%)	59 (13.8)	Male	16 (27.1) **
			Female	43 (72.9)
LVEF [%]	M (SD)	67.4 (7.8)	Male	66.4 (7.8) **
			Female	68.4 (7.6)
Systolic BP	M (SD)	135 (18.1)	Male	137.2 (18.3) *
			Female	133.1 (17.7)
Diastolic BP	M (SD)	79 (10.9)	Male	80.6 (11.6) *
			Female	78.0 (10.1)
Hypertension	N (%)	387 (91.7)	Male	187 (48.3)
			Female	200 (51.7)
Heart rate [1/min]	M (SD)	66.4 (11.4)	Male	65.4 (11.3)
			Female	67.3 (11.5)
E/A ratio	M (SD)	12.8 (4.0)	Male	12.1 (3.6) **
			Female	13.3 (4.3)
Peak VO2 [ml/kg/min]	M (SD)	16.4 (3.5)	Male	17.5 (3.4) **
			Female	15.3 (3.3)
6-min. walking test [m]	M (SD)	530.2 (87.1)	Male	551.1 (74.2) **
			Female	511.0 (93.6)
Current smoker	N (%)	27 (6.4)	Male	18 (66.7) **
			Female	9 (33.3)
Atrial fibrillation	N (%)	66 (15.6)	Male	34 (51.5)
			Female	32 (48.5)
CAD	N (%)	165 (39.1)	Male	108 (65.5) **
			Female	57 (34.5)
PAD	N (%)	17 (4)	Male	9 (52.9)
			Female	8 (47.1)
Diabetes mellitus	N (%)	70 (16.6)	Male	37 (52.9)
			Female	33 (47.1)
History of myocardial infarction	N (%)	67 (15.9)	Male	47 (70.1) **
			Female	20 (29.9)
NT-proBNP (pg/ml)	Mdn (IQR)	158.4 (299.2–83.2)	Male	150.3 (317.9–74.0)
			Female	167.6 (295.6–90.1)
SF-36 PF	M (SD)	62.6 (22.1)	Male	41.7 (9.1) *
			Female	39.3 (9.6)
HADS anxiety	M (SD)	5.3 (3.8)	Male	5.6 (3.9)
			Female	5.4 (3.6)
Social support	M (SD)	22.2 (3.8)	Male	22.2 (3.5)
			Female	22.3 (3.4)

*N* frequencies; *M* mean; *SD* standard deviation; *Mdn* median; *IQR* interquartile range; *Min* minimum; *Max* maximum; *Y* years; *NT-proBNP* N-terminal pro brain natriuretic peptide; *BP* blood pressure; *LVEF* left ventricular ejection fraction; *E/A ratio* ratio of the early (E) to late (A) ventricular filling velocities; *Peak VO2* maximum oxygen uptake; *NYHA* New York Heart Association; *HADS* Hospital Anxiety and Depression Scale; *SF-36 PF* short Form (36) Health Survey Physical function; *CAD* Coronary artery disease; *PAD* peripheral arterial disease; Social Support: ENRICHD Social Support Inventory; *: p < 0.05, **: p < 0.01 for difference between men and women (independent t-test, Chi-square-test and Mann–Whitney-U-test as appropriate);

### Correlations and multiple regression analyses between log NT-proBNP at baseline and HADS anxiety at baseline and after 12 months

Correlation analyses showed weak and non-significant associations of higher NT-proBNP at baseline with lower HADS anxiety scores at baseline and after 12 months (r = − 0.087, p = 0.09 and r = − 0.076, p = 0.15). When tested separately in the male subgroup, the association at baseline was significant (r = − 0.165, p = 0.03) and NT-proBNP at baseline also showed a marginal correlation with HADS-anxiety scores 12 months later (r = − 0.149, p = 0.06). In contrast, none of these associations were significant in female patients.

In multivariate regression models for the full cohort, the weak associations disappeared, and social support and physical function turned out to be independent predictors of low anxiety at baseline and 12 months later (Table 2).

**Table 2:** Regression analyses to predict HADS anxiety at baseline and after 12 months by baseline NT-proBNP

Dependent variable	Model	Predictor	β	p value	Corrected R^2^ (p-value)
HADS anxiety baseline	Model 1 Model 2	NT-proBNP	− 0.087	0.092	0.005 (0.092) 0.208 (< 0.001)
		NT-proBNP	− 0.010	0.848	
		Age	− 0.103	0.044	
		Social support	− 0.360	< 0.001	
		Physical function	− 0.241	< 0.001	
		Sex	− 0.010	0.830	
HADS anxiety 12 months	Model 1 Model 2	NT-proBNP	− 0.076	0.154	0.006 (0.154) 0.161 (< 0.001)
		NT-proBNP	− 0.030	0.594	
		Age	− 0.021	0.704	
		Social support	− 0.320	< 0.001	
		Physical function	− 0.259	< 0.001	
		Study arm	− 0.042	0.413	
		Sex	− 0.048	0.352	

*β* standardized regression coefficient; *p* p value; *R*^2^ coefficient of determination; *HADS* Hospital Anxiety and Depression Scale; *NT-proBNP* log-transformed N-terminal pro brain natriuretic peptide; *12 m* after 12 months; *Social Support* ENRICHD Social Support Inventory; study arm: reference = control group; Physical Function: subscale from Short Form Health Survey 36; sex: reference = male;

A similar result was observed in the male subgroup, where interestingly spironolactone treatment turned out to be an additional significant predictor for lower anxiety after 12 months (Table 3).

**Tab﻿le﻿ 3:** Regression analyses to predict HADS-anxiety at baseline and follow-up by baseline NT-proBNP for male patients

Dependent variable	Model	Predictor	β	p value	Corrected R^2^ (p-value)
HADS anxiety baseline	Model 1 Model 2	NT-proBNP	− 0.165	0.028	0.022 (0.028) 0.238 (< 0.001)
		NT-proBNP	− 0.080	0.270	
		Age	− 0.108	0.134	
		Social support	− 0.312	< 0.001	
		Physical function	− 0.320	< 0.001	
HADS anxiety 12 m	Model 1 Model 2	NT-proBNP	− 0.149	0.050	0.022 (0.058) 0.286 (< 0.001)
		NT-proBNP	− 0.068	0.365	
		Age	− 0.033	0.657	
		Social support	− 0.367	< 0.001	
		Physical function	− 0.361	< 0.001	
		Study arm	− 0.161	0.021	

*β* standardized regression coefficient; *p* p value; *R*^2^ coefficient of determination; *HADS* Hospital Anxiety and Depression Scale; *log NT-proBNP* log-transformed N-terminal pro brain natriuretic peptide; *12 m* after 12 months; *Social Support* ENRICHD Social Support Inventory; *Physical Function* subscale from Short Form Health Survey 36; study arm: reference = control group

**Fig. 1:**
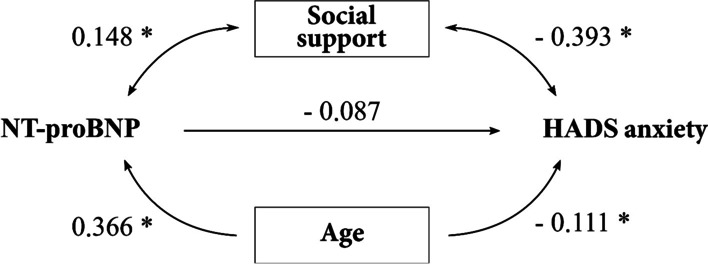
Possible mediators and confounders for the relationship between NT-proBNP and HADS anxiety. Shown are bivariate Pearson’s correlation coefficients. * = p < 0.05

Since both, social support and age correlated positively with NT-proBNP and negatively with the HADS anxiety scale in bivariate analyses (Fig. 1), we concluded that social support might either act as a mediator or as a confounder, which might influence the correlation.

**Fig. 2:**
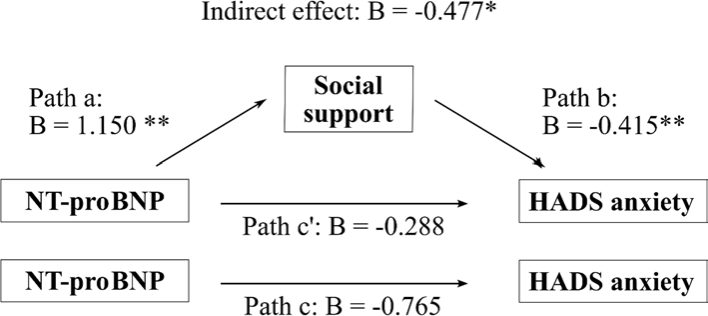
Mediation effect of social support for the association between NT-proBNP and HADS anxiety. Path a: Prediction of social support by NT-proBNP. Path b: Prediction of HADS anxiety by social support. \Path c’: Prediction of HADS anxiety by NT-proBNP with social support in the model (Direct effect). Path c: Prediction of HADS anxiety by NT-proBNP without social support in the model (Total effect). Indirect effect: Actual mediation effect by social support. * CI (-0.8770; -0.1087); ** p < 0.01

Therefore, we computed a mediation analysis with log-transformed NT-proBNP at baseline as the independent variable, HADS anxiety at baseline as the dependent variable and social support as the potential mediator. While the direct effect of NT-proBNP on HADS anxiety at baseline was not significant (B = -0.288; CI: -1.1095; 0.5334), we found that the relationship between NT-proBNP and HADS anxiety at baseline was mediated by social support with an indirect effect of -0.477 (CI − 0.8770; − 0.1087) (Fig. 2), which got weaker but stayed significant when controlled for age (B = − 0.468; CI − 0.9196; − 0.0488).

Similarly, the relationship between NT-proBNP at baseline and HADS anxiety after 12 months was mediated by social support with an indirect effect of − 0.479 (CI − 0.8676; − 0.1182), which stayed significant when controlled for age.

### Correlations and multiple regression analyses between HADS anxiety at baseline and log NT-proBNP after 12 months

The longitudinal structure of our study allowed us to examine the potential influence of anxiety at baseline on NT-proBNP levels after 12 months. Interestingly, higher HADS anxiety scores at baseline showed a clearly significant association with lower NT-proBNP values after 12 months (r = − 0.116, p = 0.03), which became even stronger when only male patients were tested (r = − 0.182, p = 0.01). In the full cohort, after multivariate adjustment for social support, age, sex, physical function and study arm, however, these associations disappeared (Table 4). The same applies if only the male patients were tested.

**Table 4:** Regression analyses to predict NT-proBNP at baseline and after 12 months by baseline HADS anxiety

Dependent variable	Model	Predictor	β	p value	Corrected R^2^ (p value)
NT-proBNP	Model 1 Model 2	HADS anxiety	− 0.087	0.092	0.008 (0.092) 0.141 (< 0.001)
		HADS anxiety	− 0.011	0.848	
		Age	0.319	< 0.001	
		Social support	0.129	0.017	
		Physical function	− 0.088	0.096	
		Sex	0.040	0.429	
NT-proBNP 12 m	Model 1 Model 2	HADS-anxiety	− 0.116	0.026	0.011 (0.026) 0.174 (< 0.001)
		HADS-anxiety	− 0.080	0.150	
		Age	0.358	< 0.001	
		Social support	0.065	0.221	
		Physical function	− 0.143	0.006	
		Study arm	− 0.036	0.459	
		Sex	0.001	0.989	

β standardized regression coefficient; *p* p value; *R*^2^ coefficient of determination; *HADS* Hospital Anxiety and Depression Scale; *log NT-proBNP* log-transformed N-terminal pro brain natriuretic peptide; *12 m* after 12 months; *Social Support* ENRICHD Social Support Inventory; *Physical Function* subscale from Short Form Health Survey 36; Study arm: reference = control group; sex: reference = male;

Mediation analyses for this association showed that the indirect effect of social support in the full cohort was not significant. For male patients, the small but initially significant indirect effect of − 0.007 (CI − 0.0169; − 0.0005) disappeared when adjusted for age.

To complement the previous results, we tested whether the data collected at six months corresponded to the associations at baseline and at 12 months. NT-proBNP at baseline significantly predicted anxiety at six months both in the full cohort (r = − 0.100; p = 0.060) and for men only (r = − 0.167; p = 0.032). When looking at anxiety at baseline as predictor and NT-proBNP as dependent variable, correlations remained negative in the full cohort (r = − 0.041; p = 0.431) with a statistical trend in the men (r = − 0.132; p = 0.079).

## Discussion

In the present study, we found weak correlations between higher NT-proBNP levels and lower scores on the HADS anxiety scale at baseline and after 12 months, which were, however, not clearly significant. This may be due to the typically observed small effect size and the somewhat smaller patient number compared to studies that found significant effects. When the male subjects were tested separately, the associations became significant or marginally significant and of similar strength as described in previous studies [12, 19] while for the female patients no significant associations were observed. Our findings are consistent with previous results concerning the association of higher plasma levels of natriuretic peptides with reduced anxiety [12, 15, 16, 19]. However, the observed correlations lost significance after adjustment for social support, age, physical function, sex and study arm, which suggests that, at least in patients with HFpEF, the mechanisms possibly linking NT-proBNP to anxiety may be more complex than originally assumed.

Correlation analyses showed that social support, as measured by ESSI, and age were positively associated with NT-proBNP levels and negatively with HADS-anxiety scores. Furthermore, mediation analysis revealed that social support acts as a full mediator, which, together with the confounding variable age, may indirectly link HADS anxiety with NT-proBNP levels. There are different explanatory approaches on how social support might act as a mediator. Possibly, BNP might exert a biological effect on the perception of social interactions leading to more trust and better self-rated social support, which in turn results in less anxiety. This effect could reflect an interaction between BNP and the neuropeptide oxytocin. Beside its main role in reproduction, social bonding and trusting behaviour, a growing body of evidence indicates that oxytocin is involved in cardiovascular regulation and fluid homeostasis [33, 34]. In an experimental model of postinfarction heart failure in rats, Wsol et al. [35] found a direct correlation between intracardiac oxytocin activation and natriuretic peptide expression. The observed heart failure four weeks after induced myocardial infarction was associated with an increase in ANP, BNP, and plasma NT-proBNP. At the same time, they observed an increase in the level of oxytocin in the muscle tissue of the right ventricle. They concluded that oxytocin exerts its cardioprotective effect in part by inducing secretion of natriuretic peptides. These results may indicate that oxytocin acts as a confounding variable that induces secretion of BNP and at the same time decreases anxiety directly or, alternatively, indirectly via a more positive perception of social support. Although a growing number of studies support a negative correlation between anxiety or stress and oxytocin levels [36, 37], study results are inconclusive and the directionality of the association is not clarified, yet. A possible explanation for an anxiolytic effect of oxytocin could be its effect on the amygdala. Higher blood oxytocin levels are associated with smaller volumes and attenuated activity in the central amygdala [38] and reduced amygdala responses to threatening social stimuli [39, 40]. Furthermore, the association seems to be moderated by gender [41]. Consequently, BNP might not have a causal effect on anxiety but could just indicate higher oxytocin levels secreted at the same time, which in turn exert an anxiolytic effect e. g. via reduced amygdala activation.

Alternatively, patients with elevated BNP due to more severe heart failure may benefit from a secondary disease gain if the disease leads to more social attention and support. In turn, greater social support may reduce symptoms of anxiety [42, 43].

Social support, together with age, might as well act as confounders that produce a spurious correlation between (NT-pro)BNP and anxiety. Possibly, persons who experience more social support might be more resilient and thus more capable of dealing with heart failure by increased secretion of BNP.

Another mechanism that could establish a causal link between natriuretic peptides, social support and anxiety is the vagus nerve. Part of the cardioprotective effect of BNP unfolds through its sympatholytic effect and the enhancement of cardiac vagal neurotransmission. BNP has been shown to enhance reflex bradycardia upon stimulation of the peripheral right vagus nerve [44] and electrical vagal nerve stimulation in rats with induced chronic heart failure resulted in an increase in LVEF and concomitant lower serum BNP [45]. In addition, results from a randomized controlled longitudinal field experiment on positive emotions through meditation suggest that an increase in positive emotions leads to higher vagotone and that this effect is mediated by a more favorable perception of social connections. Thus, the authors conclude that positive emotions, valuable social connections, and higher vagotone enhance each other in a self-reinforcing upward spiral dynamic [46]. Similarly, individuals with increased cardiac vagotone were more likely to seek social support in response to a sad experience and were less likely to experience anger. The authors explain the latter with the hypothesis that individuals with higher cardiac vagotone interpret social situations as less hostile [47]. Furthermore, there is evidence that vagus nerve stimulation reduces anxiety in treatment-resistant anxiety patients and improves quality of life in patients with heart failure [48, 49].

There is no sufficient explanation for the observed sex difference at present. In a cohort of patients with cardiovascular risk factors Sadlonova et al. [50] found a negative correlation between serum vasopressin levels and perceived anxiety only for male subjects, but not for female subjects as well. Fangauf et al. [20] found differences in men and women concerning the interaction of NT-proBNP levels and anxiety over time. Furthermore, a study of healthy undergraduates with academic stress revealed a significant interaction between cortisol elevation during exam stress and concomitant decreased NT-proBNP levels. Interestingly, only males showed a significant inverse relationship between the percent change in circulating cortisol and NT-proBNP from control to exam day. In the female group, this inverse relationship was not statistically significant [51]. The underlying mechanism for these sex differences is not known, yet, and should be addressed in future research.

The finding that treatment with the mineralocorticoid receptor antagonist (MRA) spironolactone significantly predicted lower anxiety after 12 months in the male subgroup is partly consistent with findings of previous studies suggesting an anxiolytic effect of MRAs and an anxiogenic effect of aldosterone. Long-term treatment with aldosterone in rats increased anxiety-like behavior [52] and patients with primary hyperaldosteronism tend to present with more symptoms of depression and anxiety than the general population [53–55]. In studies with both male rats [56] and patients with primary hyperaldosteronism [57], treatment with an MRA was associated with reduced anxiety. In our study, spironolactone treatment did not significantly predict lower anxiety after 12 months in the full cohort and HADS anxiety scores after 12 months did not significantly differ between the control and treatment groups. The underlying pathophysiology of the association between MRA treatment and anxiety is not entirely understood and whether the observed association in our study reflects an actual effect of spironolactone or e.g. a random significance or inclusion bias remains unclear.

Our primary research question was based on the literature suggesting that natriuretic peptides have a protective effect on perceived anxiety. However, as most previous studies in cardiac patient samples were cross-sectional only, the directionality of the observed associations was not completely clear. Interestingly, while we only observed a non-significant trend for the association between NT-proBNP values at baseline and perceived anxiety 12 months later, there was a significant negative correlation between anxiety at baseline and NT-proBNP levels 12 months later. This finding has not been reported before. Possibly, a moderate level of anxiety may have a positive effect by inducing patients to adopt a healthier lifestyle and to seek medical advice more closely, which could be associated with decreasing (NT-pro)BNP levels in the study cohort of the present study. Whether anxiety has a positive effect on lifestyle and treatment adherence in patients with chronic heart failure and is thus reflected in NT-proBNP progression should be the subject of future studies. On the other hand, increased anxiety or a possibly associated elevated stress level might have a suppressive effect on the production/secretion of (NT-pro)BNP via as yet unknown mechanisms [51]. Brouwers et al. [58] observed a negative, though non-significant, correlation between baseline HADS anxiety and NT-proBNP levels in 95 patients with systolic heart failure. In contrast to our results, their follow-up data collected 9 months later, did not reveal any significant interaction between HADS anxiety scores and NT-proBNP levels over time. Furthermore, Müller-Tasch et al. [59] did not find an association between NT-proBNP and anxiety in either the cross-sectional or the longitudinal analyses. Possible reasons for the lack of significance in these two studies might be low power due to the small sample sizes and the fact that all their patients had been diagnosed with HFrEF rather than HFpEF. Clinical severity and NT-proBNP levels were considerably higher in the Brouwers et al. and Müller-Tasch et al. studies than in ours, while previous studies showing significant associations included substantial percentages of patients with no manifest heart failure. This may indicate that with increasing severity of heart failure the association described in more mildly ill patients is lost, e.g., by downregulation of central natriuretic peptide-receptors in response to permanently elevated peptide levels. In contrast, Herrmann-Lingen et al. [11] found that high plasma levels of N-Terminal Pro-Atrial Natriuretic Peptide were associated with low anxiety in severe heart failure, as well. Further studies are needed to clarify whether our results are transferable to patients with more severe heart failure and higher NT-proBNP-levels. When more severely ill patients are included, we suggest controlling for impairments in physical function, as this could have an anxiogenic effect and thus mask a potential anxiolytic effect of BNP.

### Strengths and limitations

There are several limitations to our study findings. They were generated by means of post-hoc analyses, therefore no cause-effect relationships can be concluded from the observed associations. The majority of the patients enrolled in the ALDO-DHF study was diagnosed with NYHA Class II and less frequently NYHA Class III and was at least 50 years old. Additionally, patients whose mental state might have interfered with study adherence were excluded, as well. Consequently, our results cannot easily be generalized to younger or more severely ill patient populations. To investigate the extent to which someone suffers from anxiety, a self-report measure instead of clinical diagnostic interviews was applied. Whether NT-proBNP is related to a clinical diagnosis of anxiety disorder cannot directly be derived from our results. Although our correlation analyses between NT-proBNP and anxiety are consistent with previous findings, we could not definitely confirm them, as our results did not persist after adjustment in multiple regression analyses. Finally, due to the rather weak effects observed in our sample, it remains unclear whether they reflect real correlations or random variations. A strength is that to our knowledge, this is the first investigation of the association of NT-proBNP and symptoms of anxiety in well-characterized patients with HFpEF. Furthermore, the longitudinal nature of our data provides additional insight into the direction of the relationship between these two variables.

## Conclusions

The inconsistent associations between NT-proBNP and anxiety observed in our study and others point out that the relationship is still insufficiently understood. Interestingly, our findings provide a first hint that the association between anxiety and NT-proBNP levels may be bidirectional. More research on how cardiac-released natriuretic peptides may exert their anxiolytic effect and if anxiety impairs cardiac prognosis by suppressing counterregulatory BNP secretion is needed. Furthermore, it remains unclear how the associations between anxiety and (NT-pro)BNP levels are mediated. Therefore, potential interactions between natriuretic peptides, oxytocin, social support and anxiety need to be explored in future research, also considering possible age and sex differences.

## Data Availability

The datasets analyzed during the current study are not publicly available because they contain reidentifiable patient data and have not been approved for publication by the patients. Data are however available from the corresponding author on reasonable request.

## Abbreviations

LVEF: Left ventricular ejection fraction
HFpEF: Heart failure with preserved left ventricular ejection fraction
HFrEF: Heart failure with reduced left ventricular ejection fraction
ANP: Atrial natriuretic peptide
BNP: B-type natriuretic peptide
NT-proBNP: Amino-terminal-cleavage-fragment of B-type natriuretic peptide
DIAST-SHF: Study on prevalence and clinical course of diastolic dysfunction and diastolic heart failure
HPA axis: Hypothalamic–pituitary–adrenal axis
ACTH: Adreno-corticotropic hormone
NYHA: New York Heart Association
Aldo-DHF: Aldosterone receptor blockade in diastolic heart failure trial
HADS: Hospital Anxiety and Depression Scale
ESSI: ENRICHD Social Support Inventory
SF-36: Short Form 36 Health Questionnaire
E/A ratio: Ratio of the early (E) to late (A) ventricular filling velocities
Peak VO2: Maximum oxygen uptake
BMI: Body mass index
MRA: Mineralocorticoid receptor antagonist
